# A survey of inter-individual variation in DNA methylation identifies environmentally responsive co-regulated networks of epigenetic variation in the human genome

**DOI:** 10.1371/journal.pgen.1007707

**Published:** 2018-10-01

**Authors:** Paras Garg, Ricky S. Joshi, Corey Watson, Andrew J. Sharp

**Affiliations:** Department of Genetics and Genomic Sciences, Icahn School of Medicine at Mount Sinai, New York, New York, United States of America; University of Bristol, UNITED KINGDOM

## Abstract

While population studies have resulted in detailed maps of genetic variation in humans, to date there are few robust maps of epigenetic variation. We identified sites containing clusters of CpGs with high inter-individual epigenetic variation, termed Variably Methylated Regions (VMRs) in five purified cell types. We observed that VMRs occur preferentially at enhancers and 3’ UTRs. While the majority of VMRs have high heritability, a subset of VMRs within the genome show highly correlated variation in *trans*, forming co-regulated networks that have low heritability, differ between cell types and are enriched for specific transcription factor binding sites and biological pathways of functional relevance to each tissue. For example, in T cells we defined a network of 95 co-regulated VMRs enriched for genes with roles in T-cell activation; in fibroblasts a network of 34 co-regulated VMRs comprising all four *HOX* gene clusters enriched for control of tissue growth; and in neurons a network of 18 VMRs enriched for roles in synaptic signaling. By culturing genetically-identical fibroblasts under varying environmental conditions, we experimentally demonstrated that some VMR networks are responsive to the environment, with methylation levels at these loci changing in a coordinated fashion in *trans* dependent on cellular growth. Intriguingly these environmentally-responsive VMRs showed a strong enrichment for imprinted loci (p<10^−80^), suggesting that these are particularly sensitive to environmental conditions. Our study provides a detailed map of common epigenetic variation in the human genome, showing that both genetic and environmental causes underlie this variation.

## Author summary

Multiple published studies have demonstrated that epigenetic variation can contribute to phenotypic variation. In the present study, we identified regions of common methylation variation in five cell types, observing that these show enrichments for functional genomic features. Surprisingly, we found that these epigenetic variations can form biologically relevant networks that are specific to each cell type, often occurring near genes that have functional relevance to the cell type. Further these regions show reduced heritability, suggesting they may be responsive to environmental cues. We confirmed this by subjecting isogenic fibroblast cultures to different environmental stress. Our study provides insight into patterns of normal epigenetic variation in the human population.

## Introduction

Understanding the causes and consequences of genomic variation among humans is one of the major goals in the field of genetics. Over the past decade, studies such as the Hapmap and 1000 Genomes Projects have resulted in detailed maps of genetic variation in diverse human populations, identifying millions of single nucleotide polymorphisms, copy number variants and other types of sequence variation [1–6]. These maps have acted as the catalysts for thousands of genome-wide association studies [7], and have provided insights into diverse processes such as mechanisms of human disease, mutation, evolution, migration, selection and recombination [8–11].

However, alterations of the primary DNA sequence are not the only type of genomic variations that occur among humans. In particular there are now well-documented examples of epigenetic marks, such as DNA methylation and histone modifications, that show significant inter-individual variation [12–14]. However, in contrast to sequence polymorphism, relatively few studies have examined the distribution of epigenetic variation across the genome, and as a result our understanding of the causes and consequences of epigenetic polymorphism remains limited.

Familial and twin studies in human and mice [12,13,15–20] have shown that a substantial fraction of sites showing variable DNA methylation levels are highly heritable, and for some loci this epigenetic polymorphism has been linked with nearby genetic variation [21–24]. However, these same studies have also demonstrated that a subset of methylation variation exhibits low heritability [12,16–18,25]. While stochastic variation or technical variability could explain reduced heritability levels, differing environmental exposures such as smoking [26–28], diet/in-utero environment [29–31] and stress [32–35] have all been shown to modify the epigenome. In addition, other natural processes such as aging and X chromosome inactivation apparently underlie epigenetic variation of some sites [36–38]. Whatever the root cause of epigenetic polymorphism, several studies have demonstrated that a subset of these variations are functionally significant and associate with the expression levels of nearby genes [23,39]. Accordingly there is now substantial interest in elucidating the role of epigenetic variation in a variety of disease phenotypes [40–48], indicating that the study of epigenetic polymorphism holds significant promise for understanding the molecular etiology of disease.

In this study, we have performed a screen to identify regions of common epigenetic variation using population data derived from five different human cell types. By searching for clusters of probes with high inter-individual variability, we uncover hundreds of loci in the human genome that exhibit highly polymorphic DNA methylation levels that we term variably methylated regions (VMRs). We show that VMRs co-localize with other functional genomic features, are enriched for CpGs that influence gene expression, and provide evidence that epigenetic variability at some of these loci is influenced by both genetic and environmental factors. We also show that VMRs form *cis* and *trans* co-regulated networks enriched for transcription factor binding sites and genes with cell-type relevant functions. Finally, consistent with the notion that the epigenome represents a dynamic link between our genome and the environment[49,50], we experimentally demonstrate environmental effects on methylation at VMRs using cultured fibroblasts, revealing signatures that overlap those observed in our population-level datasets. Together, our results provide novel insights into the biology of variable methylation across the human genome.

## Results

### Identification of polymorphic DNA methylation in five human cell types

**Fig 1:**
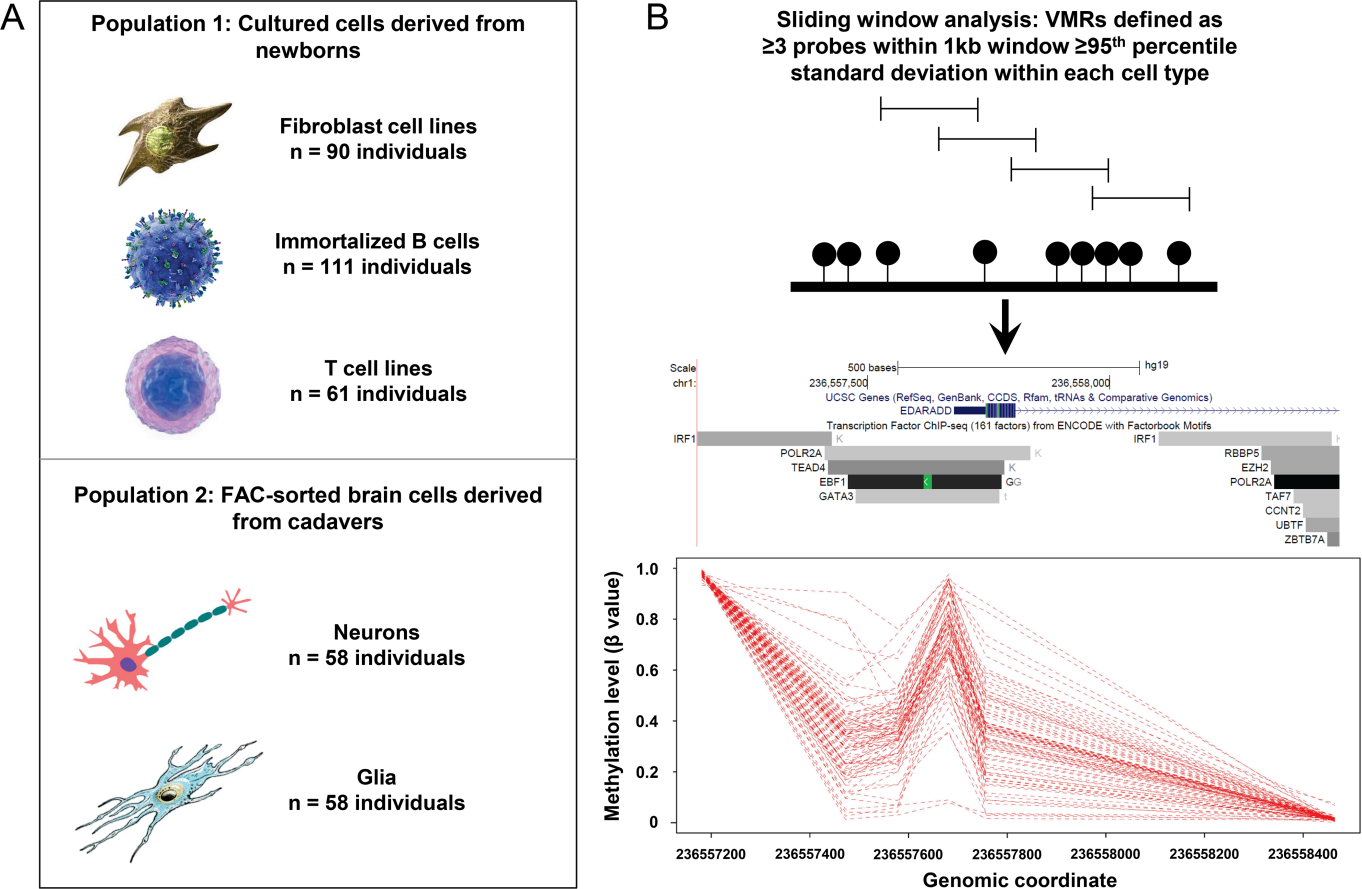
**(A)** We studied population variability of DNA methylation in five different purified cell types derived from blood, skin and brain. **(B)** Utilizing a 1kb sliding window we identified Variably Methylated Regions (VMRs), representing clusters of ≥3 probes within the top 5% of population variability within each cell type. **(C)** An example VMR identified at the promoter region of *EDARADD* in fibroblasts. As indicated by the accompanying UCSC Genome Browser tracks, ENCODE data identifies this locus as being bound by several different TFs. Dashed red lines represent DNA methylation profiles for each of the 90 cell lines from the GenCord population, showing extreme epigenetic variability at this locus in the normal population.

We performed an analysis of inter-individual variation of DNA methylation in five isolated cell types from two human cohorts (Fig 1A): 1) Primary fibroblasts, EBV-immortalized lymphoblastoid cells, and phytohemagglutinin stimulated primary T cells taken from umbilical cords of 204 newborns [23]; and 2) sorted glia and neurons from prefrontal cortical tissue from 58 deceased donors [51]. Genome-wide methylation profiles were previously generated for all samples using the Illumina Infinium HumanMethylation450 BeadChip (450k array) (Illumina, San Diego, CA, USA). After filtering (see Methods), we analyzed methylation profiles for 293,782 filtered autosomal CpGs in each of the five cell types. We utilized a sliding window approach (Fig 1B) to characterize VMRs composed of three or more neighboring CpGs with variation ≥95^th^ percentile of standard deviation of β-values in that cell type among all samples of each cell type. To avoid the confounder of gender [52], identification of VMRs was performed separately on males and females, and then the resulting set of VMRs in each gender were combined together for further analysis (see Methods).

In total, we identified 699 VMRs in fibroblasts, 1,423 VMRs in T cells, 699 VMRs in B cells, 1,137 VMRs in neuronal cells and 1,104 VMRs in glial cells. Hereafter, these VMRs are abbreviated as FVMRs, TVMRs, BVMRs, NVMRs and GVMRs, respectively. Genomic positions and relevant annotations for VMRs partitioned by cell type are provided in S1 Table. VMRs had a mean size of 863bp, and contained a mean of 6.4 CpGs (S1 Fig).

**Fig 2:**
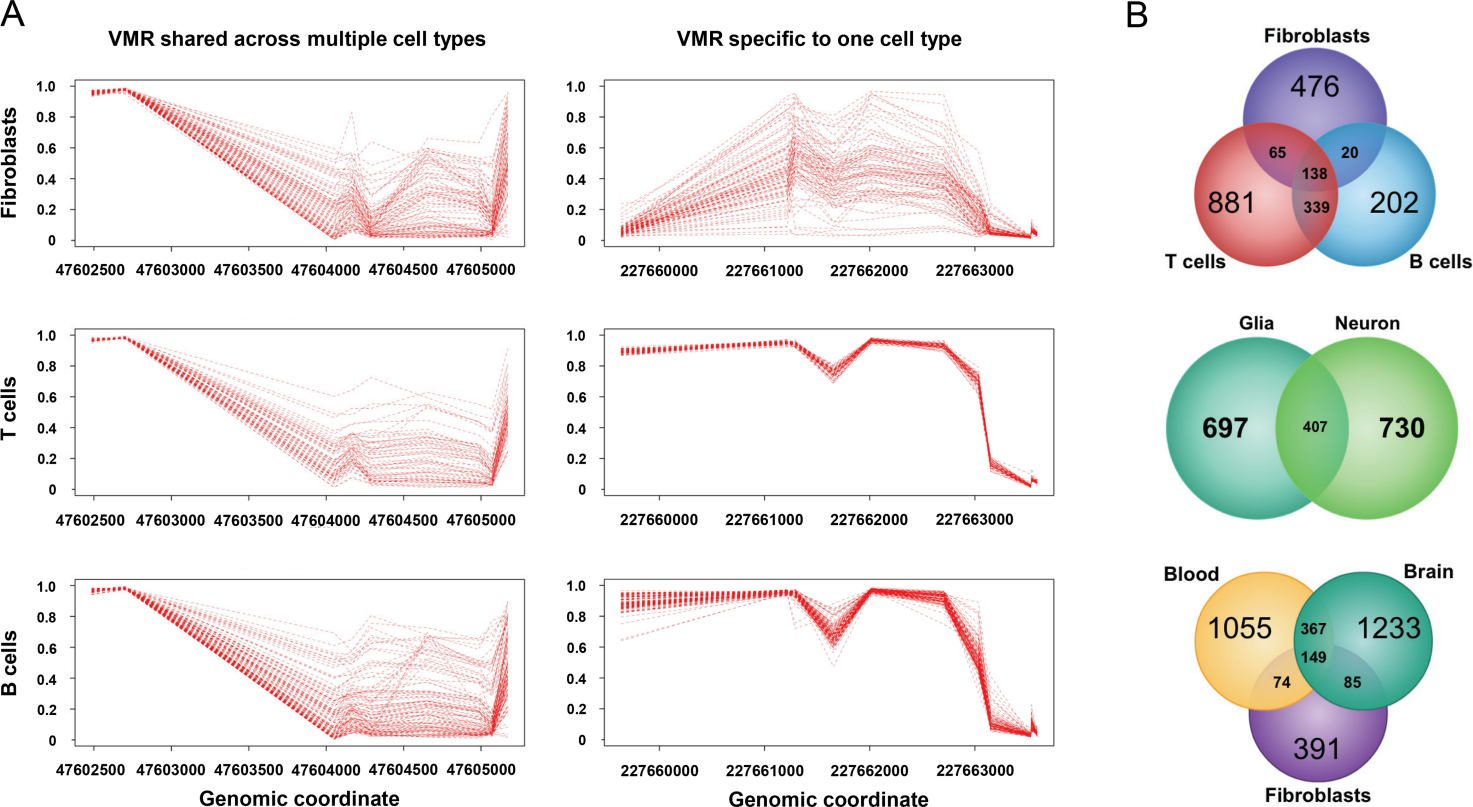
Epigenetic variation in different cell types. **(A)** While some VMRs are common to multiple different cell types, in contrast, other VMRs identified in one cell type show minimal epigenetic variation in other tissues. **(B)** Venn diagram showing the degree of overlap for VMRs found in B-cells, T-cells, Fibroblasts, Neurons and Glia.

While many characterized VMRs were specific to a given cell type, others were common across cell types and tissues. Examples of cell-type specific and shared VMRs are displayed in Fig 2A. The extent of VMR sharing between different tissues was related to their relative developmental origin. For example, approximately one third of VMRs identified in glia were also found in neurons, and ~68% of VMRs found in B cells were observed in T cells. In contrast only 23% of VMRs found in fibroblasts were also seen in B cells (Fig 2B). Between fibroblasts, blood, and brain cells, there were 149 shared VMRs (Fig 2B). In addition, by performing pairwise correlation of methylation levels at CpGs within VMRs shared in different cell types taken from the same individual, we observed much higher correlations between closely related cell types, suggesting that observed population variation is plausibly established in precursors of these cell types and maintained, or influenced by common factors and regulatory mechanisms. For example, methylation levels within VMRs shared between T cells and B cells had a mean correlation coefficient of r = 0.79 (S2 Fig). Likewise for neurons and glia, shared VMR-CpGs were highly correlated (mean r = 0.78, S2 Fig). However, the same degree of correlation was not observed for comparisons between fibroblasts and either T cells (mean *r* = 0.56) or B cells (mean r = 0.43, S2 Fig).

### Replication of VMRs in additional cohorts

Before extending our analysis of VMRs, we first replicated our approach in two additional populations. We applied our sliding window approach for identifying VMRs to (i) an additional cohort of 62 fibroblast cell lines [42,53], and (ii) a cohort of whole-blood methylomes from 2,680 individuals sampled from the general population [54]. This led to the identification of (i) 986 VMRs in fibroblasts, 230 of which were also observed in the original fibroblast population, and (ii) 1,368 VMRs in whole blood. Because this latter dataset was composed of methylation profiles generated from peripheral blood, rather than purified cell types, we compared VMRs identified in these controls with shared VMRs that were identified in both B cells and T cells in the Gencord cohort: 390 of the 477 shared B and T cell VMRs and were also found in the replication cohort, yielding a 28-fold enrichment over that expected by chance (p<2.5x10^-321^).

### VMRs preferentially overlap specific gene/CpG island features and functional elements in the human genome

Differentially methylated CpGs have been shown to often be enriched in specific regions of the genome and to co-localize with other functional epigenetic signatures [55–57]. In order to gain insight into the genomic context of CpGs in VMRs, we tested the enrichment of these CpGs in relation to various genomic features compared to a background set of CpGs assayed on the array (S2 Table).

We first performed enrichment analysis using Refseq gene and CpG island (CGI) annotations, observing consistent trends across datasets (S2 Table). Specifically, we noted that in all five of the cell types tested, VMRs were significantly enriched in 3’ UTRs and depleted in 5' UTRs (enrichments in 3’ UTRs ranging from 1.1- to 1.4-fold across the different cell types, p = 4.6x10^-2^ to p = 2.5x10^-11^). Likewise, the depletion of VMRs within 5’ UTRs ranged from 1.3- to 1.6-fold (p = 2.4x10^-8^ to p = 4.7x10^-37^) (S2 Table). The depletion in 5’ UTRs was also reflected in enrichment tests conducted using CGI annotations, which revealed significant depletions in CGIs and concomitant enrichments in CpG shores, shelves, and sea categories (S2 Table).

To further explore the co-localization of VMRs with functional genomic regions, we assessed the overlap of FVMRs and BVMRs with Chromatin State Segmentation annotations from a normal human lung fibroblast (NHLF) cell line and an EBV-immortalized lymphoblastoid cell line (GM12878), respectively; these data were previously generated by the ENCODE project [58], and included genome-wide annotations for 15 chromatin states characterized using combined epigenetic signatures from various datasets. Consistent with observed depletions in gene 5’ UTRs and CpG islands, which both tend to occur within or adjacent to gene promoters and transcriptional start sites, we also noted significant depletions of both FVMRs and BVMRs in regions defined by “Active Promoter” chromatin states in respective cell types (S2 Table). The strongest VMR enrichments in both cell types occurred in chromatin states associated with enhancer activity (S2 Table).

We also examined various other categories of genomic features in relation to VMRs, and observed the following: (i) housekeeping genes [59] were strongly under-represented in VMRs in each of the five cell types tested, (ii) loci from the GWAS catalog [60] were enriched in VMRs found in T cells, glia and neurons, (iii) loci showing human-specific methylation levels from a multi-primate analysis [61] were enriched in four of the five cell types, and (iv) loci showing parent-of-origin specific methylation associated with imprinted regions were enriched in neuronal VMRs.

### VMRs form both cis and trans co-methylated networks that are enriched for genes and transcription factor binding sites with cell-type relevant functions

**Fig 3:**
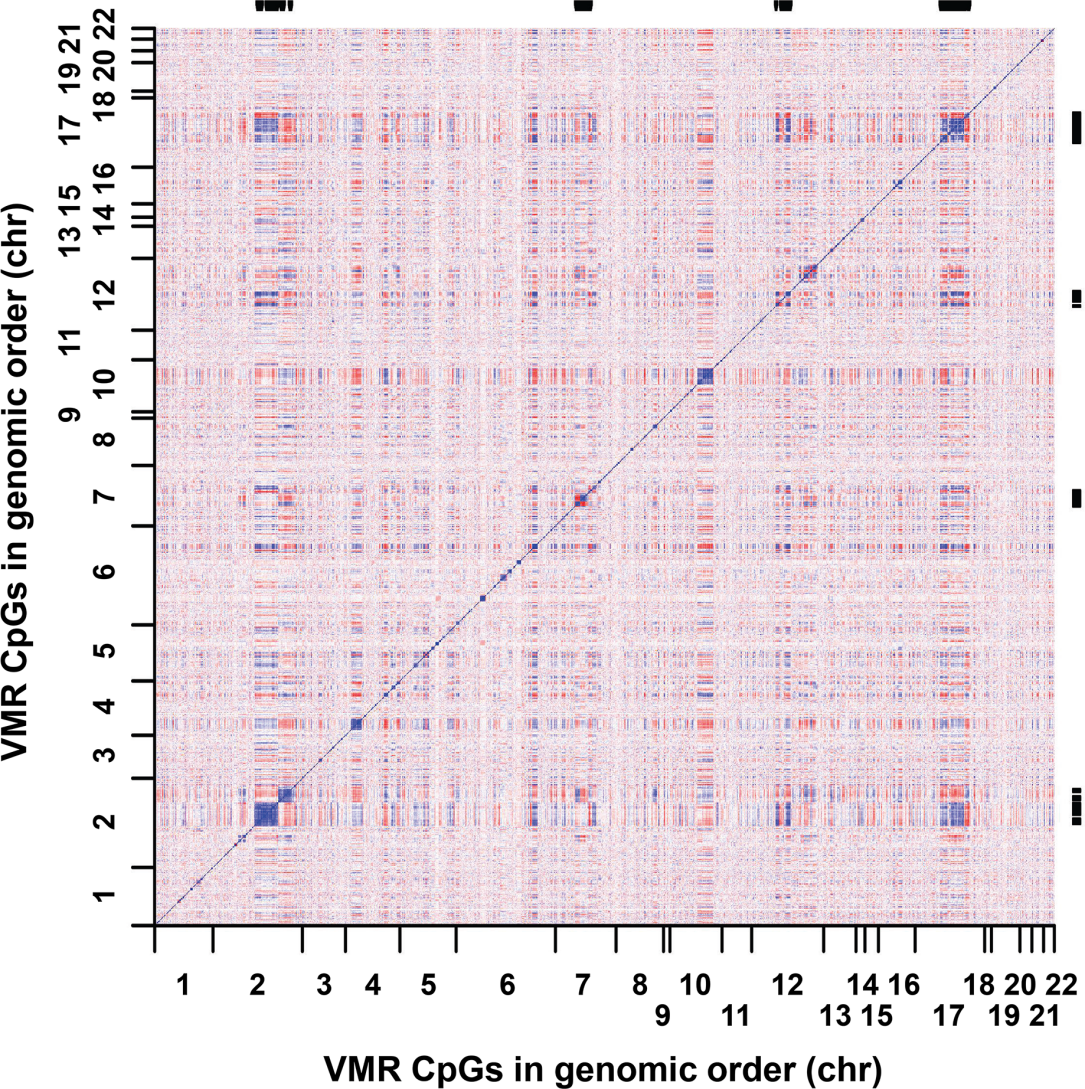
Heat map of pair wise correlation values between all CpGs located within VMRs defined in fibroblasts. CpGs on both axes are ordered by genomic position, revealing the presence of multiple VMRs located on different chromosomes that show highly correlated methylation levels in *trans*. Black bars (right and top) show the location of the *HOXA* (chr7), *HOXB* (chr17), *HOXC* (chr12) and *HOXD* (chr2) gene clusters, which correspond to some of the strongest regions of correlated methylation in both *cis* and *trans*. This observation suggests coordinated epigenetic regulation among loci distributed genome-wide.

We next sought to investigate the positional relationships of co-regulated VMRs. In each cell type we constructed pair-wise correlation matrices of all VMRs based on the β-values of the probe with the highest population variance within each VMR. The resulting heat maps of pairwise correlations revealed the presence of strongly co-methylated blocks of CpGs, whose methylation levels varied together in both *cis* and *trans*, and that these patterns were distinct to each cell type (Fig 3; S3 Fig). For example, as shown in Fig 3, FVMRs exhibit strong *cis* correlations within several chromosomal regions. Significantly, evidence of strong co-regulation *in trans* can also be seen, with several regions located on multiple different chromosomes also exhibiting strong co-variation in epigenetic state. Visual inspection of the strongest *trans* correlations in fibroblasts located on chromosomes 2, 7, 12 and 17 showed that each of these co-regulated clusters of VMRs corresponded to different members of the *HOX* gene superfamily, suggesting that such VMRs might correspond to coordinately regulated loci with shared biological functions.

**Fig 4:**
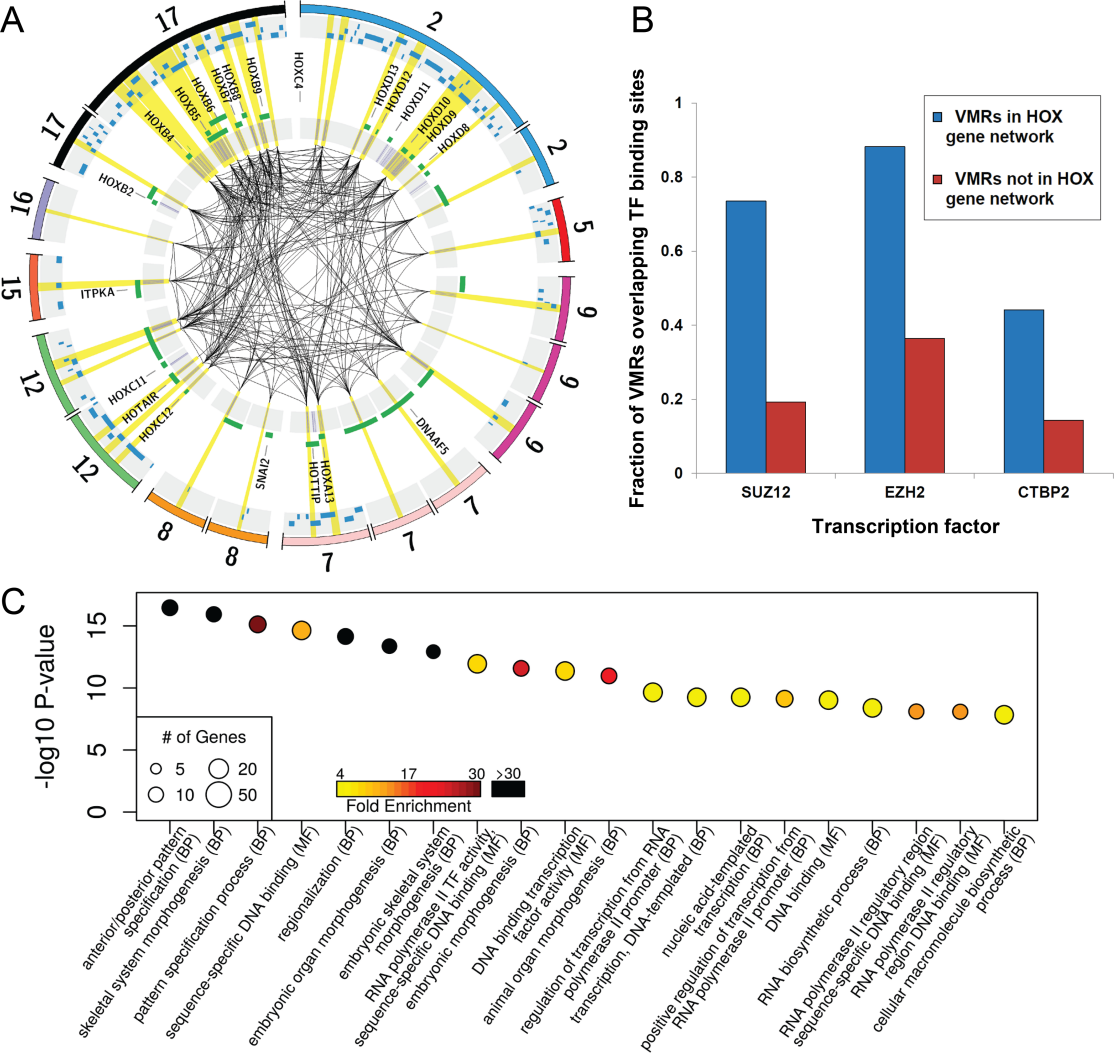
*Cis and trans* co-regulation of VMRs located at functionally related networks of genes that govern key developmental pathways. **(A)** After selecting one CpG per VMR with the highest variance, we applied WGCNA to identify networks of significantly co-regulated VMRs. The Circos plot shows a representation of one of the largest co-regulated VMR modules identified in fibroblasts, which comprises 34 independent VMRs located on four different chromosomes, comprising all four clusters of *HOX* genes (*outer circle*). CpGs within VMRs in the co-regulated module are represented by blue tick marks (*inner grey circle*), with black lines joining VMRs that have methylation levels with pair wise absolute correlation values R≥0.7 (*highlighted in yellow*). Green bars show locations of genes at each locus. Blue bars show the location of transcription factor binding sites for SUZ12, EZH2 and CTBP2, all of which are significantly enriched within this co-regulated module. **(B)** Analysis of transcription factor binding sites defined using ChIP-seq [64] showed that VMRs within the co-regulated *HOX* gene module shown in (A) are significantly enriched for SUZ12, EZH2 and CTBP2 binding compared to all VMRs defined in fibroblasts (Bonferroni corrected p = 5.3x10^-11^, p = 1.5x10^-9^ and p = 5x10^-5^, respectively). Thus, binding of these TFs represents a potential mechanism by which epigenetic variation could be coordinated at multiple independent loci in *trans*. **(C)** Results of Gene Ontology (GO) analysis of genes associated with VMRs in the most significant co-regulated module identified in fibroblasts. We identified highly significant enrichments for multiple biological processes, including body patterning, growth and morphogenesis (S5 Table).

Based on this observation, we sought to formally identify signatures of co-regulation among different VMRs. We used weighted gene co-expression network analysis (WGCNA; see Methods) [62,63], to identify co-methylated networks of VMRs within each cell type. This identified seven co-regulated modules in fibroblasts, four in T cells, two in B cells, seven in neurons, and five in glia, with each module composed of between 11 and 467 distinct co-regulated VMRs (median module size, n = 41) (S4 Table, S4 Fig). Consistent with our initial visual observations, WGCNA identified several co-regulated modules within the set of fibroblast VMRs that included all four human *HOX* gene clusters (Fig 4A).

In order to assess the biological relevance of these co-regulated VMR networks, we performed Gene Ontology (GO) enrichment analysis on the set of genes linked to the VMRs within each module (Fig 4C, S4 Table, S5 Table, and S5 Fig). Although for many networks the number of associated genes was too small to reach significance at 10% FDR, in four of the five cell types tested we identified enrichments for GO terms that were of direct functional relevance to the specific cell type. The five most significant GO enrichments and associated modules for each cell type are presented in Table 1. For example, in fibroblasts, the most significant functional categories were within the blue module that included multiple *HOX* gene clusters, including terms associated with the basic control of tissue growth and morphogenesis, such as “anterior/posterior pattern specification” (GO:0009952; 52-fold enrichment, FDR q = 3.09x10^-14^) and “embryonic organ morphogenesis” (GO:0048598; 52-fold enrichment, FDR q = 6.4x10^-12^). In T cells, the most significant GO enrichments were found for the blue module, made up of 95 co-regulated VMRs enriched for genes involved in T cell function, including the terms “T cell aggregation” (GO:0070489; 11-fold enrichment, FDR q = 1.06x10^-6^) and “T cell receptor signaling pathway” (GO:0050852; 12.8-fold enrichment, FDR q = 9.5x10^-7^). In glial cells, significantly enriched terms included a module consisting of 467 VMRs linked to genes associated with “negative regulation of neurogenesis” (GO:0050768; 3.7-fold enrichment, FDR q = 2.4x10^-4^). Finally, in neurons, the most strongly associated functional categories were with a module comprised of 18 VMRs including the GO term “synapse assembly” (GO:0007416; 61-fold enrichment, FDR q = 1.1 x10^-5^). Complete lists of enriched GO terms and modules are provided in S5 Table.

**Table 1:** The top three Gene Ontology terms associated with co-regulated VMR modules found in each cell type.

Cell Type	GO Term ID	Gene Ontology (GO) Term	Enrichment	FDR
	GO:0009952	anterior/posterior pattern specification	52.5	3.09x10^-14^
**Fibroblasts**	GO:0009952	skeletal system morphogenesis	66.1	5.20x10^-14^
	GO:0043565	sequence-specific DNA binding	10.5	5.22x10^-13^
	GO:0006955	immune response	4.8	3.24x10^-8^
**T Cells**	GO:0031295	T cell co-stimulation	24.0	9.55x10^-7^
	GO:0050852	T cell receptor signalling pathway	12.9	9.55x10^-7^
	GO:0045747	positive regulation of Notch signalling pathway	51.2	0.09261
**B Cells**	GO:0045944	positive regulation of transcription from RNA polymerase II promoter	5.3	0.09261
	GO:0008593	regulation of Notch signalling pathway	25.3	0.09354
	GO:0007156	homophilic cell adhesion via plasma membrane adhesion molecules	65.7	1.22x10^-14^
**Glia**	GO:0098742	cell-cell adhesion via plasma-membrane adhesion molecules	45.4	2.61x10^-13^
	GO:0007399	nervous system development	31.7	6.49x10^-12^
	GO:0007156	homophilic cell adhesion via plasma membrane adhesion molecules	70.1	5.61x10^-15^
**Neuron**	GO:0007399	nervous system development	27.1	5.63x10^-9^
	GO:0007416	synapse assembly	61.1	1.10x10^-5^

Based on the *trans* nature of these co-regulated VMR networks, we hypothesized that coordinated epigenetic regulation of these sites might be based on the binding of specific *trans*-acting factors to the members of each VMR network. We therefore analyzed the overlap of each VMR WGCNA module with validated transcription factor binding sites (TFBS) for 161 different transcription factors (TFs) studied by the ENCODE project [64]. We observed significant enrichments for TFBS in several VMR modules that were specific to each cell type (S6 Table). The top three enriched TFBS per cell type are provided in Table 2. In several instances, the most significant TFBS enrichments converged on modules highlighted by GO analyses. For example, EBF1 and RUNX3, which are both involved in lymphocyte differentiation and proliferation [65], were significantly enriched TFs in the blue module in T cells (RUNX3, 2.1-fold enrichment, p = 6.1x10^-7^; EBF1, 2-fold enrichment, p = 2.7x10^-5^). Similarly, in fibroblasts, TFBS for SUZ12 (3.4-fold enrichment, Fisher’s. p = 5.3x10^-11^) and EZH2 (2.3-fold enrichment, p = 1.5x10^-9^), were the most significantly enriched among VMRs of the module that included multiple *HOX*-genes (Fig 4C). Prior studies have shown that as part of the polycomb complex, SUZ12 and EZH2 have roles in the establishment of epigenetic modifications, and specifically in the regulation of *HOX* genes [66].

**Table 2:** Top 5 transcription factor binding sites overlapping with VMRs in various WGCNA modules in 5 cell types.

Cell type	TFBS	# VMRs overlapping TFBS	# VMRs in Module	Fold enrichment	p-value
	SUZ12	25	34	7.5	5.3x10^-11^
	EZH2	30	34	6.8	1.5x10^-9^
Fibroblasts	CTBP2	15	34	5.7	5x10^-5^
	TCF7L2	8	14	15.0	0.0024
	CHD1	17	52	3.0	0.012
	RUNX3	39	95	2.1	6.1x10^-7^
	ATF2	25	95	2.6	3.4x10^-6^
T cells	NFIC	25	95	2.4	8.4x10^-6^
	EP300	44	95	1.7	1.5x10^-5^
	PML	28	95	2.2	2.5x10^-5^
	RELA	12	15	5.2	2.9x10^-8^
	STAT1	7	15	6.2	3.9x10^-5^
B cells	TBP	12	15	2.7	6.6x10^-5^
	BCL3	7	15	5.4	9.1x10^-5^
	STAT3	8	15	4.4	1x10^-4^
	SUZ12	11	14	8.2	1.1x10^-9^
	EZH2	13	14	3.8	1.1x10^-7^
Glia	CTBP2	10	19	5.9	1x10^-6^
	GATA2	82	467	1.3	0.00094
	CHD1	7	14	3.8	0.00098
	CTBP2	9	32	4.2	0.00015
	HNF4G	6	22	6.0	0.00029
Neurons	EZH2	13	32	2.6	0.00047
	HNF4A	6	22	5.1	0.00071
	SUZ12	7	32	4.2	0.00086

### Methylation levels at VMRs are influenced by both heritable and non-heritable factors

**Fig 5:**
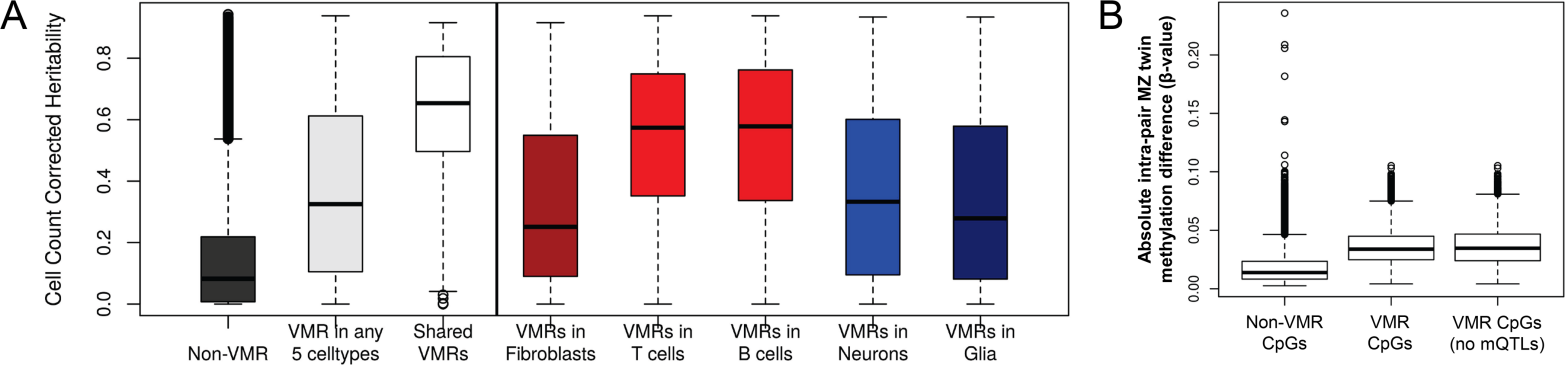
Methylation levels at VMRs are influenced by heritable and non-heritable factors. **(A)** Cell count corrected heritability [17] for VMRs in five cell types. Shared VMRs found in >1 cell type show significantly higher heritability, suggesting these are mostly under genetic control. **(B)** Methylation differences found within 426 pairs of monozygotic twins. CpGs that lie within VMRs show significantly increased MZ twin divergence compared to other CpGs, which is consistent with an environmental influence on methylation levels at VMRs.

Motivated by the signatures of co-methylation observed in our VMRs, we next sought to broadly explore the potential underlying factors associated with the regulation of VMR methylation variability. To do this, we first assessed the relationships between CpGs within VMRs, genetic variation, and gene expression. We tested for enrichment of FVMRs, BVMRs and TVMRs with previously described CpG methylation:gene expression associations (eQTMs) and CpG methylation:SNP associations (*cis* mQTLs) in fibroblasts, B cells and T cells [23]. We observed significant enrichments for VMRs in all three cell types for both CpGs that function as eQTMs and those linked with mQTLs, with enrichments of 16-, 3.4-, and 2.8-fold in eQTMs, and 4.7-, 4.8-, and 6-fold for association with mQTLs in FVMRs, BVMRs, and TVMRs, respectively (all p-values *<*10^−45^, S2 Table). To further investigate the relationship of VMRs with underlying genetic variation we used methylation heritability estimates characterized in peripheral blood leukocytes from a cohort of 117 families [19]. Overlaying heritability estimates onto VMR-CpGs across the five cell types revealed that methylation levels for CpGs within VMRs showed significantly increased heritability compared to non-VMR CpGs (Fig 5A). Thus, epigenetic variation at VMRs is often associated with nearby gene expression, and methylation levels at many VMRs shows strong evidence of being under local genetic control.

However, despite this evidence for genetic influences underlying a large fraction of epigenetic variability, the existence of co-regulated modules of VMRs *in trans* led us to hypothesize that a subset of epigenetic variation might be linked to non-genetic influences, such as differing environmental exposures. To further explore the influence of non-heritable factors on the epigenetic state of VMRs, we analyzed methylation profiles derived from whole blood samples from 426 monozygotic (MZ) twin pairs [35]. Previous studies have shown increased discordance of DNA methylation levels between MZ twins with age, presumably due to differing environmental exposures and/or stochastic processes [67]. We first identified a total of 1,289 VMRs (8,251 CpGs) in these twins (S7 Table), which showed strong overlap with VMRs identified in both B cells and T cells (64% and 59%, respectively). Based on the premise that epigenetic differences between MZ twin pairs provides a measure of the non-genetic component of epigenetic variability, at each CpG we calculated the mean absolute methylation discordance for all autosomal CpGs within each MZ twin pair. We observed a highly significant increase in MZ twin discordance for CpGs within VMRs versus non-VMR CpGs (p<10^−300^) (Fig 5B). While it is possible that this increased twin-twin discordance might be related to the inherent variability of CpGs, these observations are also consistent with the influence of environmental effects on methylation variability at a subset of VMRs.

To further investigate potential links of our VMRs with known environmental effects on DNA methylation, we utilized a published data set of Illumina 450K data generated from 128 children conceived in the rural Gambia in either the rainy or dry seasons. Here, maternal nutrition at conception shows substantial seasonal variation and is known to be associated with epigenetic differences in children conceived in each season [34]. We applied a Student’s t-test to compare methylation values in children conceived in the rainy versus dry season, calculating the resulting p-value for seasonal difference as a measure of environmental influence at each CpG. Comparing CpGs within VMRs to those in the background set of all non-VMR probes, we observed a strong enrichment for CpGs influenced by season of conception in VMRs from all five cell types when compared to the background set of p-values obtained from all 293,782 non-VMR probes (p = 8.4x10^-5^ in Fibroblasts to 1.1x10^-70^ in T cells) (S6 Fig).

### Experimental evidence for environmental influences on DNA methylation from a cell culture model

**Fig 6:**
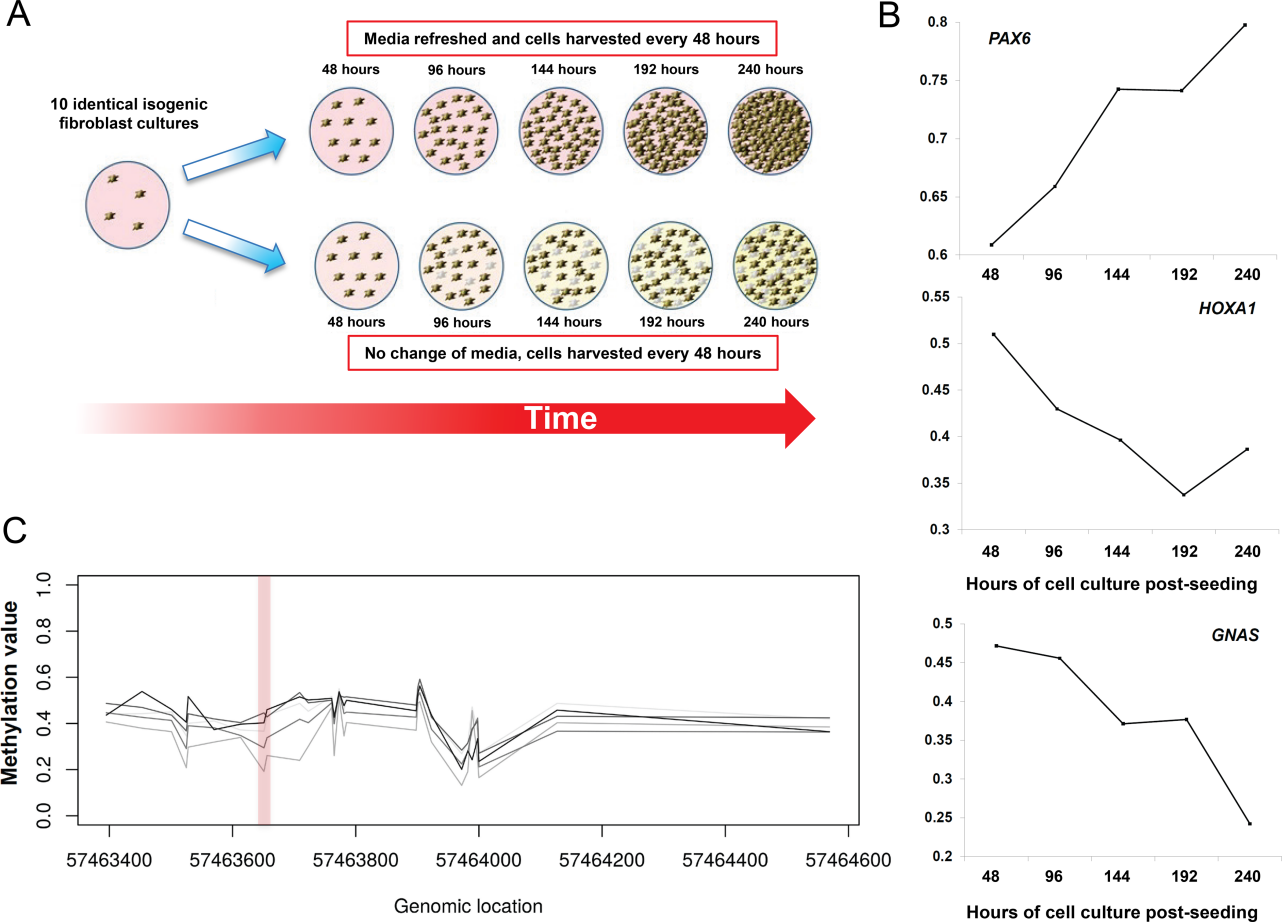
Experimental manipulation of DNA methylation using cell culture shows enrichment for VMRs at *HOX* genes and imprinted loci. **(A)** To directly assess the effect of varying environmental conditions on epigenetic state, we grew genetically-identical fibroblasts under conditions of varying cell density and culture media replenishment. Cells from a single human fibroblast line were seeded in parallel at low density in ten culture flasks, and allowed to grow continuously for up to 10 days, either with or without regular change of media. Every 48 hours one flask was harvested and genome-wide DNA methylation patterns profiled. **(B)** Applying a sliding window approach identified 135 VMRs where methylation levels showed robust changes with varying culture conditions, including loci at *HOX* genes and multiple imprinted loci. Gene ontology analysis of VMRs induced by cell culture showed enrichments for fundamental control of growth, including similar GO categories to the co-methylated network identified in fibroblasts from the Gencord cohort (S9 Table). **(C)** Environmentally-responsive VMRs induced by cell culture showed a 35-fold enrichment for probes within the differentially methylated regions associated with seven different imprinted genes (p = 5.6x10^-79^). The left plot shows methylation profiles at the imprinted region of *GNAS*, which was also identified as a VMR in cultured fibroblasts. Each line shows the methylation profile at a different time point, with lighter shades of grey with increasing time. The right plot shows the change in methylation level with time at a single CpG (cg09885502) within the *GNAS* VMR.

To experimentally verify whether methylation levels at some VMRs are responsive to environmental cues, we performed cell culture experiments in which we grew genetically identical fibroblasts under different environmental conditions, varying the rate of culture media replenishment and cell density with time (summarized in Fig 6A). Skin fibroblasts from a single normal male (GM05420) were seeded in parallel from a single master culture into eight separate flasks, and allowed to grow under normal or low-nutrient conditions, achieving varying levels of cell density at each time point. Every 48 hours one flask was harvested from each media replenishment regime, DNA extracted and profiled on the 450k array, resulting in DNA methylation profiles for nine samples (see Methods).

We reasoned that as these fibroblast cultures shared an identical genetic background, any epigenetic variations observed among them would be attributable to non-genetic factors, such as varying culture environment. We applied the same sliding-window method to identify VMRs in methylation data from these cultured isogenic fibroblasts, identifying 135 putatively “environmentally responsive” VMRs. This included many of the same VMRs identified previously in our population-based analysis of umbilical cord-derived fibroblasts (S8 Table), with a 5.3-fold enrichment for overlap between these two sets of VMRs compared to the background set of probes on the array (p = 2.9x10^-29^). Examples of VMRs showing changes in methylation level with culture conditions are shown in Fig 6B.

Concordant with our population analysis, GO analysis of the 135 VMRs from cultured isogenic fibroblasts revealed enrichments for *HOX* genes, as well as several of the same GO terms associated with the co-regulated FVMR modules (S9 Table). Strikingly, these environmentally responsive VMRs were also enriched 35-fold for CpGs within known imprinted loci versus the null (p = 5.6x10^-79^). This included overlaps with differentially methylated regions associated with the imprinted genes *MKRN3*, *IGFIR*, *ZNF331*, *PEG3*, *L3MBTL*, *GNAS* and *MEST* (Fig 6C) [68].

## Discussion

Here we surveyed variation in DNA methylation patterns in five purified human cell types, identifying hundreds of genomic loci that exhibit a high degree of epigenetic polymorphism in the human population: we term these ‘Variably Methylated Regions’ or VMRs. We observed that VMRs are enriched for various functional genomic features, most notably enhancers, suggesting a potential role in regulating gene expression patterns. Unexpectedly, we found that many VMRs form co-regulated networks both *in cis* and *in trans*, with multiple VMRs spread across different chromosomes at which methylation levels vary in a coordinated fashion. These co-regulated networks were specific to each cell type, had reduced heritability, and were also enriched for gene sets with cell-type relevant functions. For example, we observed VMR networks associated with genes enriched for synaptic transmission in neurons, regulation of nervous system development in glia, and T cell activation in T cells. These observations suggest that some VMRs represent loci that form co-regulated pathways that are implicated in the regulation of genes with cell-type specific functions. The dispersed nature of these co-regulated VMR networks indicates that they are potentially regulated by *trans* acting factors, and consistent with this we found significant enrichments for relevant transcription factor binding sites associated with some networks.

While many VMRs are influenced by local genotypes, our analyses of monozygotic twins, a cohort of African samples conceived in divergent nutritional environments, and *in-vitro* culture of genetically identical fibroblasts cell lines indicates that epigenetic variation at some VMRs is linked to environmental factors. Indeed, using isogenic fibroblast cultures derived from a single individual that were grown under different environmental conditions, we were able to replicate many of the same VMRs found in our original population analysis, thus showing that epigenetic variation at these loci is an environmentally inducible trait. Intriguingly these environmentally-responsive VMRs showed a strong enrichment for imprinted loci (p<10^−94^), suggesting that these genes are particularly sensitive to environmental conditions. This observation that varying cell culture conditions result in epigenetic alterations across the genome, presumably accompanied by changes in gene expression, highlights that the use of cultured cells for investigating epigenetic phenomena should be approached with caution. We suggest that unless carefully controlled, variations in cell culture conditions could easily introduce significant epigenetic and transcriptional changes that could confound many *in vitro* studies.

VMRs in fibroblasts comprised co-regulated modules that included all four *HOX* gene clusters that are each located on different chromosomes. While a previous study has reported that the *HOX* genes exhibit variable methylation that correlates with their expression levels [53], our analysis builds on these observations by showing that methylation across multiple *HOX* gene clusters is correlated in both *cis* and *trans*. Furthermore, using validated transcription factor binding sites, we found a significant enrichment for transcription factors EZH2 and SUZ12 at these VMR sites associated with *HOX* genes. These two transcription factors are components of the Polycomb Repressive Complex 2 (PRC2), which functions as a histone H3K27-specific methyltransferase and regulates both epigenetics and expression of *HOX* genes [66]. Thus, we propose a model where coordinated variation of DNA methylation at multiple loci in *trans*, corresponding to a network of co-regulated genes, is under the control of transcription factor binding in response to physiological and/or environmental cues. In the case of the *HOX* gene network in cultured fibroblast cell lines, such cues could be the availability of nutrients, local cell density and other growth conditions, allowing the cells to modify their growth trajectories in response to the prevailing environment. Consistent with this model, recent observations were made in macrophages, a type of immune cell that has a variety of roles in different tissues around the body, which mirror our findings. Two prior studies showed that the epigenetic state of enhancer elements in these cells responds to the tissue microenvironment in which they reside, and is regulated by networks of tissue- and lineage-specific transcription factors that drive divergent programs of gene expression [69,70]. Studies of chromatin accessibility have also shown that manipulating the presence of specific transcription factors can lead to global modification of epigenetic state at multiple loci *in trans* [71].

We defined VMRs as clusters of probes with high variance, as the use of single probes to determine epigenetic variability is inherently unreliable. This is because other phenomena unrelated to epigenetic variance can influence the β-value reported by a probe, independent of DNA methylation levels. These include, for example, underlying genetic variants that alter probe binding, random technical effects such as hybridization or wash artifacts on the array, or the simple fact that some probes might simply perform poorly and yield inherently more variable results. By considering groups of multiple closely spaced probes that all show high variability makes it much less likely that our results would be influenced by the effects listed above, thus improving specificity for the detection of true epigenetic variability, and reducing artifacts.

One of the strengths of this study is that we specifically utilized purified cell types for our analysis, some of which were also of homogeneous age. This has the advantage of removing the confounder of both cellular heterogeneity and age effects, both of which are known to influence DNA methylation [36,37,72]. Such differences would otherwise result in many false positive VMRs due to underlying differences in cell fractions or age among individuals.

One of the limitations of this analysis is that we used methylation profiles from the Illumina 450k array, which targets only a small subset (~3%) of CpGs in the human genome, and has coverage that is biased towards gene promoters and CpG islands. As such, the maps of VMRs we provide here are far from comprehensive, and future work that utilizes more comprehensive approaches (*e*.*g*. whole genome bisulfite sequencing) will undoubtedly provide more complete genomic maps of epigenetic variation. However, to our knowledge currently no such datasets on a population-scale are available. One other potential caveat is that the methylation profiles for B cells, fibroblasts and T cells were all generated from cells that had been cultured *in vitro*, and furthermore the B cells were also immortalized by Epstein-Barr virus infection, a process which is known to induce widespread epigenetic changes [73]. However, we observed good replication of the VMRs identified from cultured/immortalized B cells and T cells in an independent cohort where DNA was extracted from uncultured blood, indicating that many of these same VMRs observed even in immortalized B cells are also present natively.

One confounder that deserves mention is that there is potential that some of the enrichments we identify between VMR probes and factors such as mQTLs and eQTMs could be driven by the inherent variability of VMRs. This is because statistical power to identify an association with a locus is heavily influenced by the underlying variability of that site. Thus, it is possible that some of enrichments we observe are due either partly or wholly to this effect. Similarly, it is also possible that the increased discordance we observed in MZ twin pairs at VMRs might be driven simply by the higher variability of methylation at these loci.

In conclusion our study of DNA methylation polymorphism provides novel insights into the nature and function of epigenetic variation. The coordinated phenomenon we observed where methylation levels at networks of multiple genomic regions varies in response to the local environment is consistent with popular theories that the epigenome can indeed act as an interface between the genome and environment [39,50,74].

## Materials and methods

### Data processing and statistical analysis

We obtained DNA methylation data generated using the Illumina 450k HumanMethylation BeadChip from two published studies. We utilized data from the Gencord cohort from the EMBL-EBI European Genome-Phenome Archive (https://www.ebi.ac.uk/ega/) under accession number EGAS00001000446, representing 90 fibroblast cultures, 61 T-cell cultures and 111 immortalized B-cell cultures derived from a cohort of newborns [23]. We also utilized methylation data representing FAC-sorted glial and neuronal cells from 58 deceased donors downloaded from GEO (http://www.ncbi.nlm.nih.gov/geo/) under accession number GSE41826 [51]. Prior to analysis for methylation variation, each dataset underwent several filtering and normalization steps, as follows. In each individual, probes with a detection p>0.01 (mean n = 348 per sample) or mapping to the X or Y chromosomes were removed. 482,421 probe sequences (50-mer oligonucleotides) were remapped to the reference human genome hg19 (NCBI37) using BSMAP, allowing up to 2 mismatches and 3 gaps, retaining those 470,576 autosomal probes with unique genomic matches. Probe coordinates were converted to hg19 using *liftover*. Probes that overlapped SNPs identified by the 1000 Genomes Project (minor allele frequency ≥0.05) either including or within 5bp upstream of the targeted CpG (n = 13,376 autosomal probes) were discarded, as such variants can introduce biases in probe performance. We also removed probes overlapping copy number variants of ≥5% frequency in CEU HapMap samples [75].

After filtering, we retained 457,200 autosomal and 11,021 chrX probes on which we performed a two color channel signal adjustment and quantile normalization on the pooled signals from both channels and recalculation of average β-values as implemented in the *lumi* package of R [76]. The Illumina Infinium HumanMethylation450 BeadChip contains two assay types (Infinium type I and type II) which utilize different probe designs. As the data produced by these two assay types shows distinct profiles, to correct this problem we performed a beta mixture quantile normalization method utilizing BMIQ [77] on the normalized data. β-values were quantile normalized using the normalizeQuantile function in the *aroma*.*light* R package. One pair of neuronal/glial samples was excluded on the basis that they showed discrepant gender, as determined by PCA analysis of β-values on the sex chromosomes. We further removed 22,267 probes where any of the cell types had >5% probes with detection p-value >0.01. For sliding window analysis, we sub-selected 293,782 autosomal 1kb windows containing 3 or more probes. CpGs in these regions were then annotated based on their position relative to RefSeq genes using BEDTools v2.17 [78].

### Pairwise correlation analysis

To compare similarity of VMRs across cell types, we utilized the fact that >1 cell type was available from each individual (Fibroblasts, T cells and B cells in one cohort, and Neurons and Glia in the other). We compared the similarity of VMRs between two cell types by first selecting probes from VMRs found in both cell types, and then computing pairwise Pearson correlation coefficients on the β-values from the two cell types from each individual. The number of individuals and probes in pairwise VMRs were:

1. FVMR-BVMR: 57 individuals, 725 probes
2. BVMR-TVMR: 58 individuals, 1886 probes
3. FVMR-BVMR: 87 individuals, 553 probes
4. NVMR-GVMR: 58 individuals, 1740 probes

### Variably methylated regions

To identify regions of common highly variable methylation that should be robust to fluctuations in single probes, we chose an approach to identify loci containing multiple independent probes showing high population variance. To avoid gender effects creating false positives in our analysis, either biological or technical due to cross-hybridization artifacts [52], we first divided males and females and analyzed each gender separately. For each probe, we calculated the standard deviation (SD) of the β-value separately in each cell type. We then utilized a 1kb sliding window based on the start coordinate of each probe, beginning at the most proximal probe on each chromosome and moving down consecutively to the last probe on each chromosome. We defined VMRs as those 1kb regions containing at least 3 probes ≥95^th^ percentile of SD in that cell type, with an additional criterion that at least 50% of the probes in that window were also ≥95^th^ percentile of SD. The relevant scripts used for this paper can be found at Github (https://github.com/AndyMSSMLab/VariableMethyl). VMRs that were found in the same cell type in either males or females were then combined, and used in all downstream analysis.

### Network analysis and gene ontology analysis

To identify potential co-regulation relationships among VMRs, we applied Weighted Gene Correlation Network Analysis (WGCNA) to each set of VMRs identified per cell type [63]. Input values for each VMR were β-values for the variable probes within VMR which had standard deviation ≥95^th^ percentile. As suggested in the WGCNA user manual, we plotted scale-free topology fit indices and mean connectivity plots with varying soft-thresholding power. Based on these plots in all five cell types, we chose power of 6 as our soft-thresholding power and scale free topology fit index>0.8 in all cases. We generated adjacency matrices by raising the correlation matrix to the power of 6, which was then transformed into topological overlap matrix (TOM). VMRs were then classified into modules using hybrid dynamic tree cutting with a minimum cluster size of 10 VMRs. VMRs in each module were selected at Module Membership value ≥0.7. We associated VMRs with gene annotations based on either their localization within ±2kb of Refseq transcription start sites, overlap with DNAseI hypersensitive sites that showed significant association *in cis* with gene expression levels within ENCODE cell lines [79], and significant associations between methylation and gene expression levels (eQTMs) in T cells, B cells, fibroblasts [23]. The fraction of VMRs that were linked to a gene varied from a low of 78.4% in T cells, to a high of 81.8% in Fibroblasts. If two VMRs were members of the same module, but located on different chromosomes, then this was considered a *trans* association, *cis* otherwise. For each module we performed Gene Ontology enrichment analysis using in house scripts. Each VMR was annotated with Refseq genes which either overlapped gene body or promoter region as described above. Refseq genes associated with 293,782 probes tested where used as background for gene ontology analysis. P-values for each GO term were generated using the hypergeometric distribution and incorporated 5% FDR correction.

### Enrichment analysis of transcription factor binding sites

We downloaded the track of Uniform transcription factor binding sites (TFBS) from the UCSC Genome browser [80], containing experimentally determined binding sites for 162 transcription factors. As the precise boundaries of some VMRs were not well defined, we extended TFBS coordinates by ±500bp prior to overlap with the set of VMRs identified in each cell type. Enrichment analysis for TFBS to occur within each module of co-regulated VMRs identified by WCGNA versus the background was performed using a Fisher's exact test. The 2x2 table for Fisher’s exact test contained whether the probe is in a specific module or not and whether they overlap TFBS or not.

### Replication of VMRs in whole blood

Methylation dataset for Replication study (2,680 samples) was downloaded from GEO (GSE55763) and was normalized the same way as described above [54]. VMRs were called with same criteria. A VMR in discovery cohort was considered successfully replicated if the VMR coordinates in discovery cohort overlap (minimum 1 bp) with VMRs found in replication cohort.

### Publicly available datasets for enrichment analyses

We downloaded dataset for CpG features, Refseq gene annotations (3’ UTR, 5’UTR, CDS, Intron & Intergenic), GWAS catalog, Segmental Duplications, Simple Repeats and ChromHMM features from the UCSC table browser [80]. eQTMs and mQTLs were obtained from Gutierrez-Arcelus M et al. [23]. Sites showing human-specific methylation patterns from an analysis of multiple primate species was obtained from Hernando-Herraez et al. [61]. 647 genes thought to be linked with environmental response were obtained from NIEHS website (https://www.niehs.nih.gov), and 1,847 genes categorized under the GO term “response to environmental stimulus” were obtained from Amigo [81]. Cell-count corrected heritability estimates for CpG methylation were obtained from McRae et al. [19], and annotations of CpG islands showing evolutionary constraint and biased gene conversion (BGC) from Cohen et al. [82].

All enrichment analyses were performed by overlapping probe coordinates in VMRs with respective feature and using all 293,782 probes as background. The enrichment p-value was generated using Hypergeometric distribution (phyper function in R). The fold enrichment was calculated using following formula: (probes in VMR overlapping Feature/probes in VMR)/(probes in Background overlapping Feature/probes in Background).

### Assessment of environmental effects using MZ twins

We downloaded published data set of Illumina 450K data generated from 430 monozygotic (MZ) (GEO dataset GSE105018) [35], and 128 children conceived in the rural Gambia in either the rainy or dry seasons (GSE99863) [34]. After normalization using the same methods as described above, we applied a Student’s t-test to compare methylation values in children conceived in the rainy versus dry season, and calculated the resulting p-value for seasonal difference, a measure of this environmental effect. A Wilcoxon Rank-Sum test was performed to compare the distribution of this p-value between VMRs and non-VMRs.

### Fibroblast cell culture and methylation profiling

A growing culture of human skin fibroblasts from a normal male individual (GM05420) was obtained from Coriell Institute for Medical Research (Camden, NJ). Cells were grown in RPMI1640 media supplemented with 1mM L-glutamine, 10% FBS and 100u/L each of penicillin and streptomycin. A single vial of fibroblasts was initially grown in a 2ml culture plate, with media changed every 24 hours. Once the cells attained 80% confluency they were trypsinized and split equally into two T25 flasks. Each flask was treated identically, with media changed every 24 hours until the cells achieved 80% confluency (approximately 7 days after seeding). Both cultures were then trypsinized, mixed, and the cells seeded equally into a total of nine T25 flasks, which were then harvested at set time points (TP) under different culture regimes, as follows:

1. Harvested immediately
2. Time Point (TP) 1—harvested after 48 hours
3. TP2—fresh media given at TP1 and then harvested after a further 48 hours
4. TP3—fresh media given at TP1 and TP2, and then harvested after a further 48 hours
5. TP4—fresh media given at TP1, TP2 and TP3, and then harvested after a further 48 hours
6. TP5—fresh media given at TP1, TP2, TP3 and TP4, and then harvested after a further 48 hours
7. TP2a –No change of initial media, harvested after 96 hours
8. TP4a –No change of initial media, harvested after 192 hours
9. TP5a –No change of initial media and then harvested after 240 hours

At each time point, cells were harvested by trypsinization, pelleted by centrifugation, and frozen at -20 Celsius. Once all cultures were harvested, DNA was extracted in a single batch using the Qiagen DNeasy blood and tissue kit and these samples processed together on a single chip using the Illumina 450k HumanMethylation BeadChip according to manufacturer’s instructions. The resulting data were then processed and normalized as described above. Given small sample size, we excluded the step where we performed quantile normalization on β-values (aroma.light), and VMRs across these nine samples defined as 1kb regions containing at least 3 probes ≥95^th^ percentile of SD, with an additional criterion that at least 50% of the probes in that window were also ≥95^th^ percentile of SD. Methylation array data from cell line GM05420 have been deposited in the NCBI Gene Expression Omnibus (GEO) (http://www.ncbi.nlm.nih.gov/geo) under accession number GSE76836.

## Supporting information

S1 Fig Distribution of number of CpGs and length of VMR in five cell types. VMRs contained a mean of 6.4 CpGs, with average size of 863bp.
(TIF)

S2 Fig Pairwise correlation between shared VMRs reveals varying levels of similarity across cell types. To measure similarity in the distribution of VMRs between cell types, we performed pairwise correlation of methylation levels at CpGs within VMRs shared in different cell types taken from the same individual. VMRs found in fibroblasts show relatively low correlations with other cells types, whereas there is much greater similarity in VMRs between T-cells and B-cells (both of which are types of blood cell), and even greater similarity between VMRs found in glia and neurons (both of which are derived from brain).
(TIF)

S3 Fig Heat maps showing pair wise correlation values between all CpGs located within VMRs defined in neurons, glia, B cells and T cells. In each plot, CpGs on both axes are ordered by genomic position, revealing the presence of multiple loci located on different chromosomes that show highly correlated methylation levels in *trans*.
(TIF)

S4 Fig Examples of networks of genes associated with co-regulated VMRs identified in four cell types. The outermost circle in each Circos plot represents segments of each chromosome. Gene names are shown inside. Blue tick marks on the inner grey shaded band represent each VMR. The central black curved lines connect VMRs in the network that have methylation levels with pair wise absolute correlation values R≥0.7.
(TIF)

S5 Fig Results of Gene Ontology (GO) analysis of genes associated with networks of co-regulated VMRs identified by WCGNA. **(A)** T cells, **(B)** glia, and **(C)** neurons. No significant enrichments were detected in B cells.
(TIF)

S6 Fig Boxplot of association with seasonal nutrition shows significant enrichments for environmental effects within VMRs. Boxplots show the -log_10_ p-value for t-test between β-values for children conceived in the rural Gambia in the rainy versus dry season. The dotted horizontal line corresponds to the median of non-VMR probes to allow visual comparison across the categories. Below the x-axis are show p-values from a Wilcoxon Rank Sum test comparing the distribution of p-values in each boxplot with the background distribution.
(TIF)

S1 Table VMRs defined in neurons, glia, B cells, T cells and fibroblasts. (XLSX)

S2 Table Enrichment analysis for various genomic features overlapping VMRs in five cell types. (XLSX)

S3 Table Networks of co-regulated VMRs defined by WCGNA in neurons, glia, B cells, T cells and fibroblasts. (XLSX)

S4 Table Genes associated with networks of co-regulated VMRs defined by WCGNA in neurons, glia, B cells, T cells and fibroblasts. Based on the Networks of co-regulated VMRs defined by WCGNA (Supplementary S3 Table), VMRs were associated with the genes they regulate based on either their localization within ±2kb of transcription start sites, overlap with DNAseI hypersensitive sites that showed significant association *in cis* with gene expression levels (Sheffield et al., 2013), and significant associations between methylation and gene expression levels (eQTMs) in T cells, B cells, fibroblasts (Gutierrez-Arcelus et al., 2013) and monocytes (Liu et al., 2013).
(XLSX)

S5 Table Significantly enriched Gene Ontology (GO) categories associated with genes linked with networks of co-regulated VMRs in neurons, glia, B cells, T cells and fibroblasts. For each module with at least 10 constituent genes, we performed Gene Ontology enrichment analysis using GOrilla (Eden et al., 2009).
(XLSX)

S6 Table Significantly enriched transcription factor binding sites overlapping networks of co-regulated VMRs defined by WCGNA in neurons, glia, B cells, T cells and fibroblasts. (XLSX)

S7 Table VMRs defined by analysis of methylation in 426 individuals, representing 213 pairs of monozygotic twins. (XLSX)

S8 Table VMRs identified in cultured isogenic fibroblasts grown under conditions of increasing cell density and nutrient deprivation. (XLSX)

S9 Table Results of GO enrichment analysis using genes associated with VMRs identified in cultured isogenic fibroblasts grown under conditions of increasing cell density and nutrient deprivation. (XLSX)
