# Supplementary figures and images for: A survey of inter-individual variation in DNA methylation identifies environmentally responsive co-regulated networks of epigenetic variation in the human genome
Source: PLoS Genet. 2018 Oct 1;14(10):e1007707. doi: 10.1371/journal.pgen.1007707 (PMC6181428; doi:10.1371/journal.pgen.1007707)

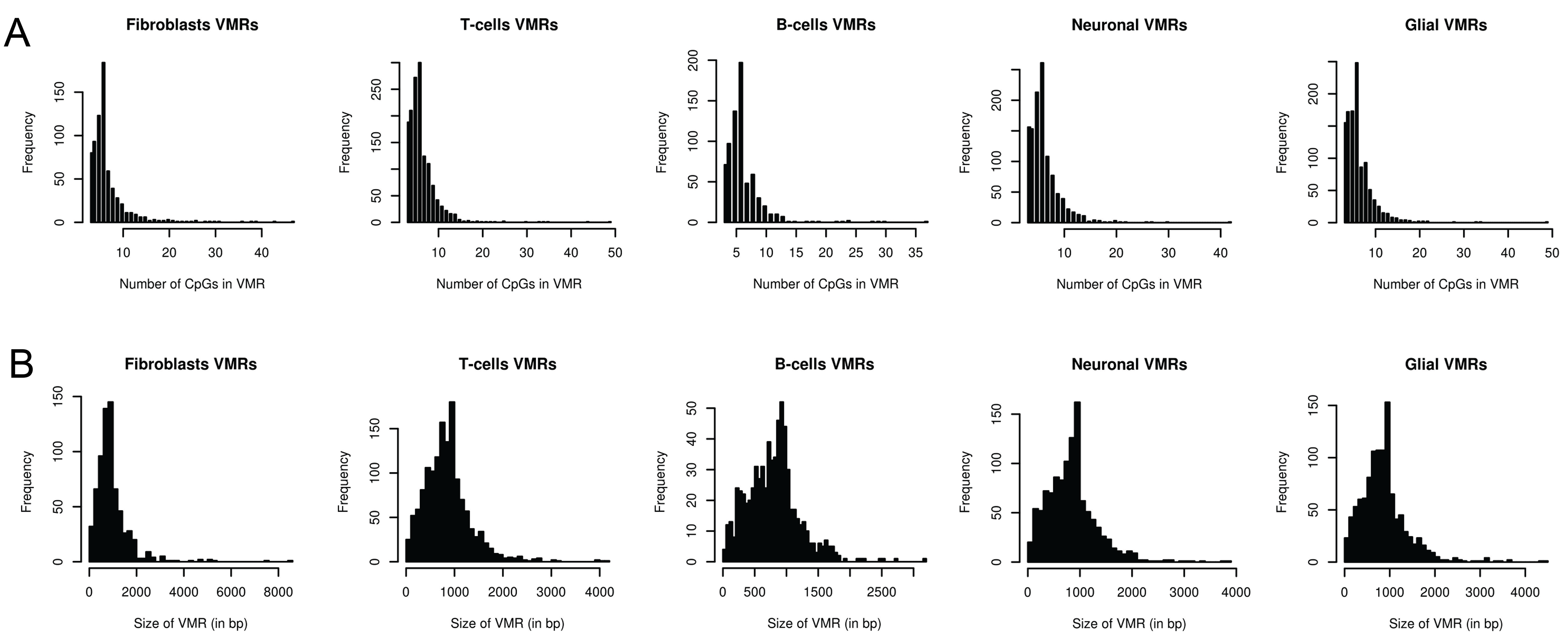

Supplement: S1 Fig — VMRs contained a mean of 6.4 CpGs, with average size of 863bp. (TIF) [file pgen.1007707.s001.tif]

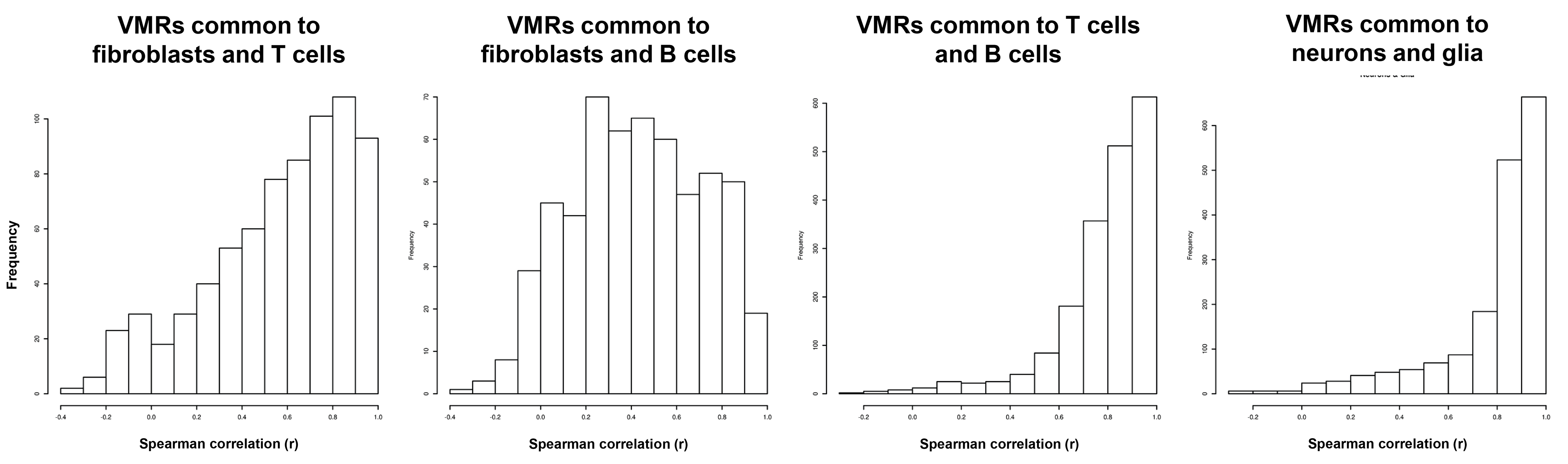

Supplement: S2 Fig — To measure similarity in the distribution of VMRs between cell types, we performed pairwise correlation of methylation levels at CpGs within VMRs shared in different cell types taken from the same individual. VMRs found in fibroblasts show relatively low correlations with other cells types, whereas there is much greater similarity in VMRs between T-cells and B-cells (both of which are types of blood cell), and even greater similarity between VMRs found in glia and neurons (both of which are derived from brain). (TIF) [file pgen.1007707.s002.tif]

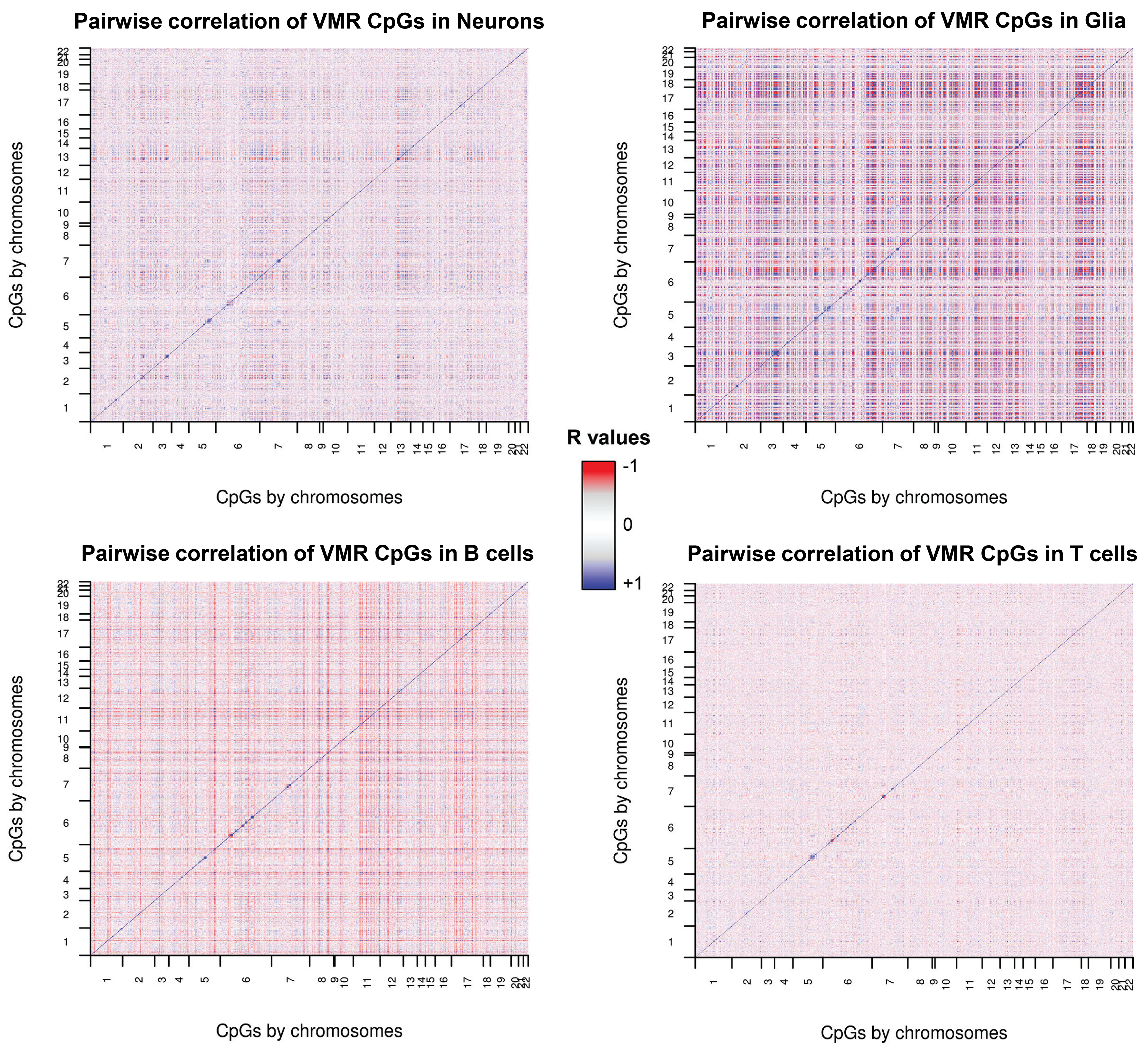

Supplement: S3 Fig — In each plot, CpGs on both axes are ordered by genomic position, revealing the presence of multiple loci located on different chromosomes that show highly correlated methylation levels in trans. (TIF) [file pgen.1007707.s003.tif]

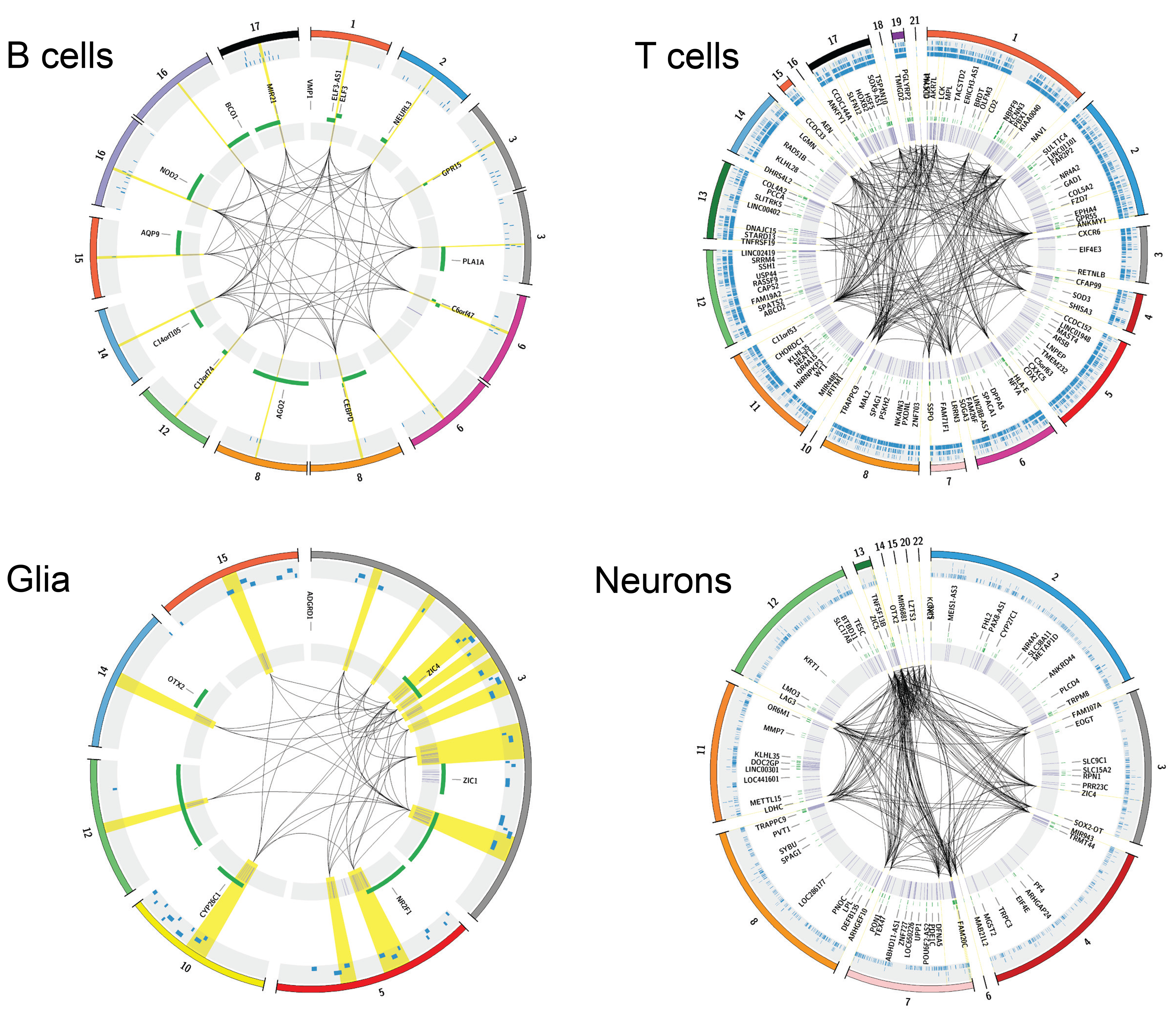

Supplement: S4 Fig — The outermost circle in each Circos plot represents segments of each chromosome. Gene names are shown inside. Blue tick marks on the inner grey shaded band represent each VMR. The central black curved lines connect VMRs in the network that have methylation levels with pair wise absolute correlation values R≥0.7. (TIF) [file pgen.1007707.s004.tif]

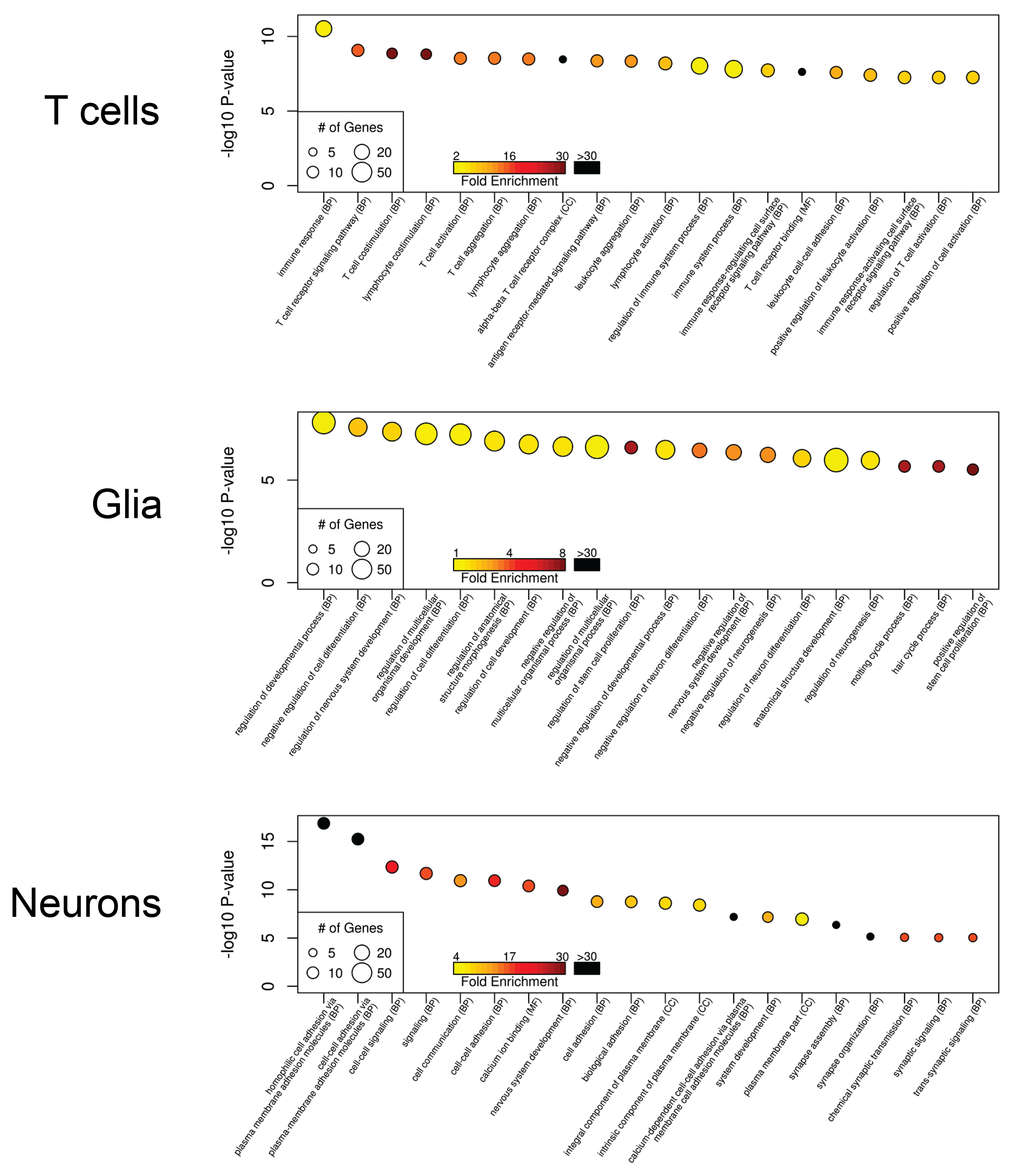

Supplement: S5 Fig — (A) T cells, (B) glia, and (C) neurons. No significant enrichments were detected in B cells. (TIF) [file pgen.1007707.s005.tif]

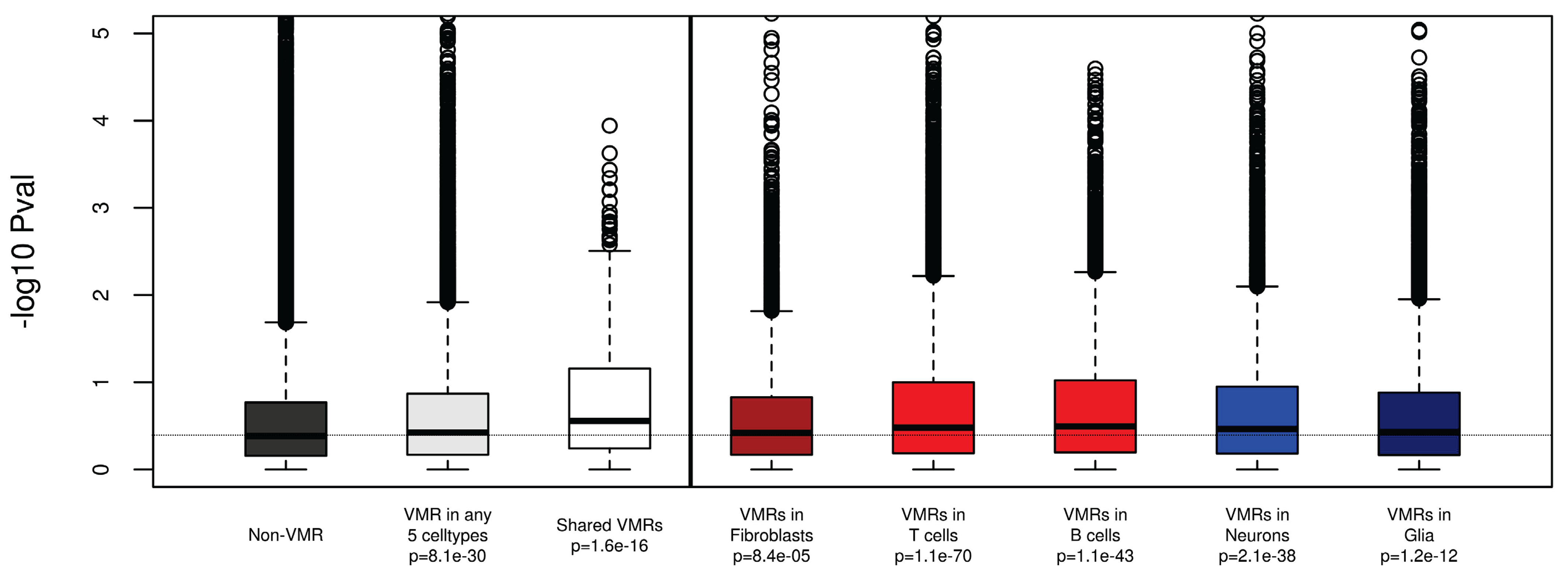

Supplement: S6 Fig — Boxplots show the -log10 p-value for t-test between β-values for children conceived in the rural Gambia in the rainy versus dry season. The dotted horizontal line corresponds to the median of non-VMR probes to allow visual comparison across the categories. Below the x-axis are show p-values from a Wilcoxon Rank Sum test comparing the distribution of p-values in each boxplot with the background distribution. (TIF) [file pgen.1007707.s006.tif]
